# Rapid and reliable diagnostic method to detect Zika virus by real-time fluorescence reverse transcription loop-mediated isothermal amplification

**DOI:** 10.1186/s13568-018-0591-6

**Published:** 2018-04-18

**Authors:** Xu-Guang Guo, Yong-Zhuo Zhou, Qin Li, Wei Wang, Jin-Zhou Wen, Lei Zheng, Qian Wang

**Affiliations:** 1 Laboratory Medicine Center, Nanfang Hospital, Southern Medical University, Guangzhou, 510515 Guangdong Province China; 2 Department of Clinical Laboratory Medicine, The Third Affiliated Hospital of Guangzhou Medical University, Guangzhou, 510150 Guangdong Province China; 3 Department of Clinical Laboratory Medicine, The Third Affiliated Hospital of Southern Medical University, Guangzhou, 510150 Guangdong Province China; 4 Section of Pulmonary, Critical Care and Sleep Medicine, Department of Medicine, Yale School of Medicine, Yale University, New Haven, CT 06520-8057 USA; 5 Department of Laboratory Medicine, The First Affiliated Hospital of Shenzhen University, Shenzhen, 518035 Guangdong China; 6 Department of Laboratory Medicine, Shenzhen Second People’s Hospital, Shenzhen, 518035 Guangdong China; 7 Center For Disease Control and Prevention of Chaozhou, Guangzhou, 521000 Guangdong Province China

**Keywords:** Zika virus, NS5 gene, LAMP, Loop-mediated isothermal amplification, Real-time fluorescence reverse transcription

## Abstract

To detect Zika virus more rapidly and accurately, we developed a novel method that utilized a real-time fluorescence reverse transcription loop-mediated isothermal amplification (LAMP) technique. The NS5 gene was amplified by a set of six specific primers that recognized six distinct sequences. The amplification process, including 60 min of thermostatic reaction with *Bst* DNA polymerase following real-time fluorescence reverse transcriptase using genomic Zika virus standard strain (MR766), was conducted through fluorescent signaling. Among the six pairs of primers that we designate here, NS5 was the most efficient with a high sensitivity of up to 3.3 ng/μl and reproducible specificity on eight pathogen samples that were used as negative controls. The real-time fluorescence reverse transcription LAMP detection process can be completed within 35 min. Our study demonstrated that real-time fluorescence reverse transcription LAMP could be highly beneficial and convenient clinical application to detect Zika virus due to its high specificity and stability.

## Introduction

Zika virus is primarily transmitted by the bite of the female *Aedes aegypti* mosquito in tropical regions, which must feed on blood to lay eggs (Gulland 2016; Walsh 2016). Other species of mosquito have been reported such as *Aedes polynesiensis* and *Aedes albopictus*. The virus has also been isolated from a number of arboreal mosquito species in the *Aedes* genus.

Patients with active or recessive infection and non-human primates infected with Zika virus are the source of the disease. The symptoms of infection are similar to dengue fever and include fever, rash, joint pain, muscle pain, headache and conjunctivitis (Interlandi 2016).

Only 20% of infected people present clinical symptoms, including fever, rash, joint pain and conjunctivitis. The symptoms usually disappear in less than a week. However, Zika virus could pose serious problems for pregnant women and their fetus. Zika can be transmitted to the fetus in the uterus or during the delivery process (Chen and Tang 2016). Zika virus nucleic acid has been detected in milk. In early 2015, a widespread epidemic of Zika fever, caused by the Zika virus in Brazil, spread to other parts of South and North America. The incidence in China is relatively low but the number is steadily increasing. Zika virus infection has become a serious global public health threat to humans (Worobey 2017). The outbreak period is usually associated with the mosquito breeding season. Thus, a fast and efficient method to detect Zika virus is necessary.

Real-time loop mediated isothermal amplification (LAMP) is a novel technology that has been developed in recent years (Xia et al. 2014). LAMP is a thermos-stable nucleic acid amplification technique for gene detection that requires 4–6 primers targeting 4–6 specific sequences (Mansour et al. 2015). It only takes 60 min for the *Bst* DNA polymerase to amplify the target sequences. LAMP technology has been widely used in detecting a variety of viruses, bacteria and parasites, which has been useful for pathogen identification in clinics (Khan et al. 2012).

Compared with traditional serologic test, LAMP is fast and efficient and may be a particularly useful method for infectious disease diagnosis in low and middle-income countries. LAMP does not require expensive equipment, and the process can be performed with a water bath or incubator (Hattori et al. 2016). Thus far, detection methods for Zika virus have primarily relied on traditional real-time polymerase chain reaction (RT-PCR). In this study, we demonstrated that Zika virus can be accurately detected by LAMP and that it is less time-consuming and has a higher sensitivity than traditional RT-PCR, suggesting that this method could be beneficial tothe clinical treatment and prevention of Zika infections.

## Materials and methods

### Zika virus and bacterial strains

The nucleic acid of Zika virus MR766 RNA was purchased from Beijing Ruilimaidi Laboratory Co., Ltd. *Klebsiella pneumoniae ATCC700603*, *Acinetobacter baumannii ATCC19606*, *Streptococcus pneumoniae ATCC49619*, *Staphylococcus aureus ATCC25923*, *Streptococcus mitis ATCC49456*, *Pseudomonas aeruginosa ATCC27853*, *Haemophilus influenzae ATCC49766* and *Escherichia coli ATCC25922* were stored in the microbiology room of the Third Affiliated Hospital of Guangzhou Medical University.

### Primer design and synthesis

The primers forward outer primer (F3), backward outer primer (B3), forward internal primer (FIP), backward internal primer (BIP) and loop backward primer (LB) were designed by Guangzhou Deaou Biotechnology Company Limited and synthesized by Tiangen Biochemical Technology (Beijing) Company Limited. The sequences of the primers used in this study are listed in Tables 1, 2, 3 and 4.

**Table 1:** The primer NS5-1 used for detection of Zika virus

Primers	Sequence (5′ → 3′)	Length (bp)
F3	GCTATGACTGACACCACAC	19
B3	ACCTTGGATCATTCACAGC	19
FIP (F1c + F2)	CCAGCTCCTTCCACAGCCCAAGAAGGTACTCGCCAG	36
BIP (B1c + B2)	TAAGCGGCCACGTGTCTGAATATTGCTCCCAGTGCTG	37
LoopF	AGGAAGCGACCATGTTCATT	20
LoopB	GAAGAGTTCATCAACAAGGTGC	22

**Table 2:** The primer NS5-2 used for detection of Zika virus

Primers	Sequence (5′ → 3′)	Length (bp)
F3	CATCTTCCCTCGTGAATGG	19
B3	ACCTTGTTGATGAACTCTTCTT	22
FIP (F1c + F2)	CGGTGTGGTGTCAGTCATAGCGGTTGTTAGACTCCTGTCAAA	42
BIP (B1c + B2)	GACACCAGGGTGCCAGACGGAAGCGACCATGTTCAT	36
LoopF	CCTGTAACTCCAGTCACCAC	20
LoopB	AGAAGGTACTCGCCAGGT	18

**Table 3:** The primer NS5-3 used for detection of Zika virus

Primers	Sequence (5′ → 3′)	Length (bp)
F3	GCCAACAAAGAGTCTTCAAAG	21
B3	ACAACTATGGCACTCTCCT	19
FIP (F1c + F2)	AGACACGTGGCCGCTTACGCCAGGTAATGAACATGGT	37
BIP (B1c + B2)	GCAGCACTGGGAGCAATATTTGACCTTGGATCATTCACAGC	41
LoopF	CCTTCCACAGCCAGGAAG	18
LoopB	AATGGAAGACGGCTGTGG	18

**Table 4:** The primer NS5-4 used for detection of Zika virus

Primers	Sequence (5′ → 3′)	Length (bp)
F3	TCTTCCCTCGTGAATGGG	18
B3	ACCTTGTTGATGAACTCTTCTT	22
FIP (F1c + F2)	CGGTGTGGTGTCAGTCATAGCTTGTTAGACTCCTGTCAAAGC	42
BIP (B1c + B2)	GACACCAGGGTGCCAGACGGAAGCGACCATGTTCAT	36
LoopF	CCTGTAACTCCAGTCACCAC	20
LoopB	AGAAGGTACTCGCCAGGT	18

### LAMP reaction system preparation

Bacterial genomic DNA extraction kit was purchased from Tiangen Biochemical Technology Company Limited. The LAMP reaction system was prepared according to the manual for the LAMP amplification kit.

### Primer screening and dissociation curve analysis

Four sets of primers were designed: NS5-1, NS5-2, NS5-3, and NS5-4. Each of the working fluids to Zika virus RNA served as a template. The reaction procedure was set to 63 °C 15 s, 63 °C 45 s as one cycle for 60 cycles, 95 °C 15 s to terminate the reaction, and 63 °C 45 s at the collection of fluorescence signals. A reaction tube was used for the dissolution curve detection and to compare the amplification efficiency of four sets of primers while screening out the highest amplification efficiency without a primer dimer.

### Sensitivity of LAMP for Zika virus

The RNA concentration of Zika virus was measured by adjusting the RNA template concentration with 1 μl of TE buffer to the Thermo Scientific Nanodrop 2000 microphotometer and 1 μl of RNA extract of Zika virus. 10 μl of RNA stock solution was diluted 10-fold with ultra-pure water four times to obtain five RNA template concentrations. This was added to the LAMP system reaction, and the amplification curve was observed to evaluate the sensitivity of the primers.

### Specificity and repeatability of LAMP for Zika virus

The genomic DNA of Zika virus and other negative pathogens were extracted and amplified according to the reaction conditions of Real-LAMP. The specificity of the primers was evaluated. One of the positive strains and five negative strains were tested three times with LAMP. The same primer used for the repeat test was used to evaluate the reliability of LAMP.

## Results

### Primer screening test of LAMP assay

**Fig. 1:**
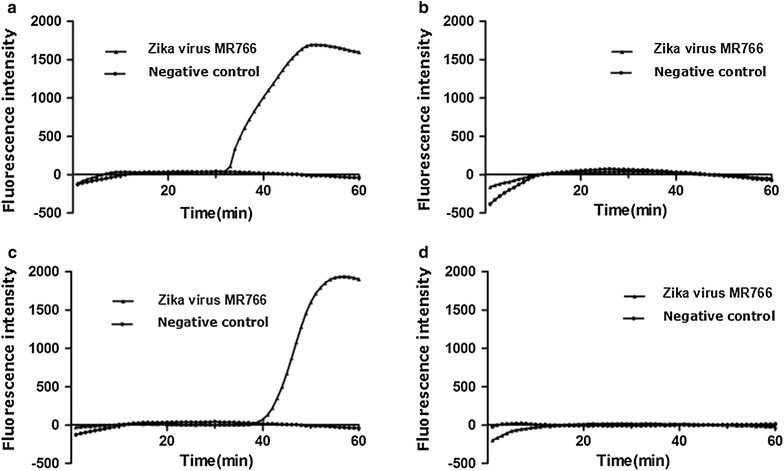
The primer of the LAMP screening test in this study: **a** real-amp without loop primers (NS5-1); **b** real-amp without loop primers (NS5-2); **c** real-amp without loop primers (NS5-3); **d** real-amp without loop primers (NS5-4)

**Fig. 2:**
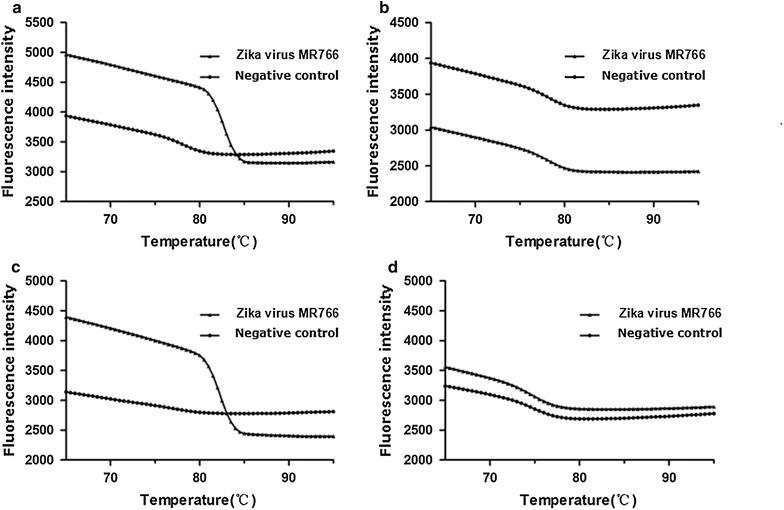
The LAMP melting curve in this study: **a** real-amp without loop primers (NS5-1); **b** real-amp without loop primers (NS5-2); **c** real-amp without loop primers (NS5-3); **d** real-amp without loop primers (NS5-4)

Four sets of primers amplified without the loop primer showed that the primers NS5-3 had a peak at about 39 min and that the NS5-1 primers had a peak at about 31 min. The efficiencies are both lower than those with the loop primers. The NS5-2 and NS5-4 primers had no peak (Figs. 1, 2).

**Fig. 3:**
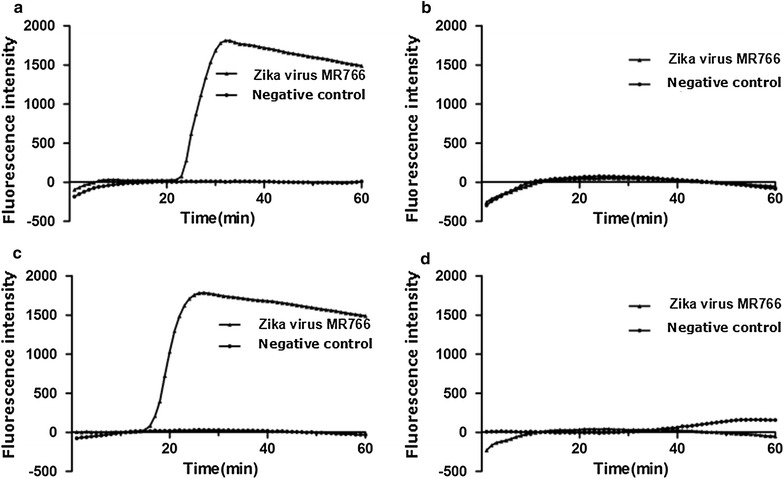
The primer of LAMP screening test in this study. **a** Real-amp with loop primers (NS-1); **b** real-amp with loop primers (NS5-2); **c** real-amp with loop primers (NS5-3); **d** real-amp with loop primers (NS5-4)

**Fig. 4:**
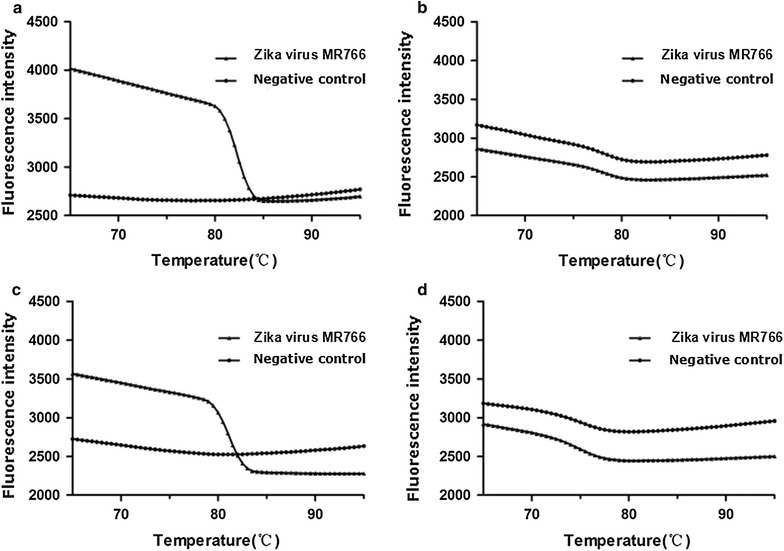
The LAMP melting curve in this study. **a** Real-amp with loop primers (NS5-1); **b** real-amp with loop primers (NS5-2); **c** real-amp with loop primers (NS5-3); **d** real-amp with loop primers (NS5-4)

At approximately 15 min after the initiation of the reaction, the system of the primer NS5-3 peaked, and at approximately 21 min, the reaction of the NS5-1 primers peaked. The Bio-Rad CFX Manager detected the amplification signal of the NS5 gene, NS5-2 and NS5-4 without a peak (Fig. 3). Although the primer NS5-3 had a relatively high amplification efficiency for the Zika virus NS5 gene, the fluorescence intensity of the negative control curve was significantly decreased, possibly for the dimer of primers (Fig. 4).

### Sensitivity of LAMP for amplification of Zika virus

**Fig. 5:**
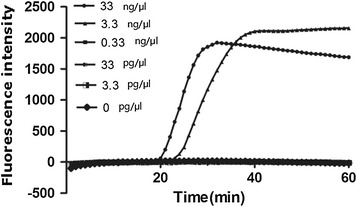
Sensitivity of LAMP for the amplification of Zika virus

In this study, the clinical common pathogens were selected as negative controls, but no Zika virus RNA was amplified. The typical “S” curve showed that the specificity of Zika virus RNA was better. The LAMP primer NS5-1 has good specificity and does not cross-react with non-target bacteria (Fig. 5).

### Specificity of LAMP for amplification of Zika virus

**Fig. 6:**
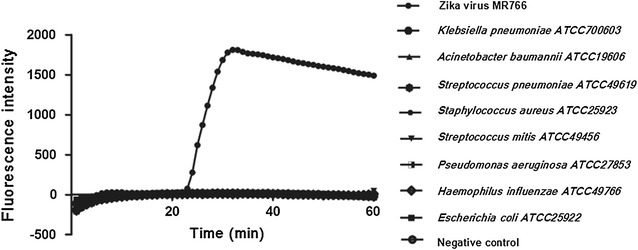
Specificity of LAMP for the amplification of Zika virus

Measured by micro-spectrophotometer, the experiment’s Zika virus nucleic acid concentration is 33 ng/μl. Diluted by 4 gradients, the resulting concentrations were 3.3, 0.33 ng/μl, 33 and 3.3 pg/μl. The limit of the detection of this test can reach 3.3 ng/μl. Reliable test results are shown in Fig. 6.

### Repeatability of LAMP for amplification of Zika virus

**Fig. 7:**
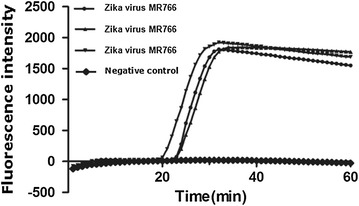
Repeatability of LAMP for the amplification of Zika virus

Take Zika virus NS5 for three replicates, wherein two peaks were at 23 min and another peak was at 20 min. The difference in peak time is approximately 3 min. The repeatability of the tests is shown in Fig. 7.

## Discussion

The Zika virus belongs to the *Flaviviridae* family and the *Flavivirus* genus and is thus related to the dengue fever, yellow fever, Japanese encephalitis, and West Nile viruses (Zuanazzi et al. 2017). Most patients show no symptoms or have mild symptoms (Zhang et al. 2017). Only 20% of patients experience fever, rash, conjunctivitis and joint pain, while in some cases, symptoms disappear within a week (Wilbe et al. 2017; Zamani and Zamani 2017). Therefore, to develop a rapid and robust nucleic acid amplification assay to efficiently detect Zika virus is critically important for clinical treatment and disease prevention.

In this study, the NS5 gene was selected as the target gene and 8 clinical common pathogens were used as negative controls. The yellow fever virus was theoretically selected as the negative control.

NS5 gene sequences were successfully amplified and displayed as a typical ‘S’ shape in Zika virus, except for other negative pathogens, which were used as negative controls, indicating that the NS5 gene sequence as a highly specific candidate could be used in Zika detection. The results showed that the ring primer could significantly shorten the reaction time. The reaction of the ring primer lasted approximately 22 min, and the peak time of the ring primer was less than 10 min. The entire reaction process can be completed within 35 min, which is significantly shorter than that for PCR and is conducive to the clinical diagnosis of Zika virus infection, the timely detection of Zika virus infection, and timely treatment.

Traditional PCR methods are based on agarose gel electrophoresis, which are cumbersome to operate (Wong et al. 2017). Our current method, the LAMP amplification technology, is superior due to its high sensitivity and specificity and ease of operation. Generally, the amplification could be visualized and quantified by fluorescence dye intensity. Primers were specifically labeled with fluorescent dye and embedded in the amplified double-stranded DNA. False positive results could be obtained by the dimer in the primer. In our study, we carefully checked the dissolution curve for the NS5 primer. The negative control curve was straight while the positive control curve was nearly overlapped, indicating that the NS5 primer contained no dimer.

LAMP detection technology has the following advantages compared with other methods. The primers used in the LAMP detection technique are based on six primers designed for the target sequence (Abdul-Ghani et al. 2012; Ahmad and Hashsham 2012; Deborggraeve and Buscher 2012; Ebenezer et al. 2012; Njiru 2012). In addition, LAMP is superior to other methods due to its high sensitivity, as high as 3.3 ng/μl. No positive signals were observed in the negative control. These results were reproducible, and we repeated them three times in our study with no significant difference in peak time. An important feature of the LAMP method is the very short detection time and small amount of reagents needed. LAMP is a consistent reaction system that avoids temperature changes, which may influence accuracy.

However, there are limitations. For example, inevitable aerosol pollution may lead to false positive results (Asiello and Baeumner 2011; Fu et al. 2011). LAMP is less common than PCR and is primarily used as a diagnostic or detection technique, not for cloning (Beck and Henrickson 2010). In addition, the LAMP reaction system requires more primers, which may increase the possibility to form dimers (Gill and Ghaemi 2008; Mori and Notomi 2009).

Our current study demonstrated that the application of LAMP to detect Zika virus was superior to traditional methods with a high sensitivity and specificity. As a rapid and efficient novel method, LAMP may be a beneficial tool for clinical application.

## Abbreviation

LAMP: loop-mediated isothermal amplification
